# Children’s birth weight and the risk of general obesity and central obesity in primary school children: a 4-year longitudinal study

**DOI:** 10.3389/fpubh.2025.1469226

**Published:** 2025-03-24

**Authors:** Yi Lin, Richard Rankin, Stuart McDonald, Xiao-Yong Li, Feng Wang, Si-Jia Wang, Qing-Hai Gong, Feng Tong

**Affiliations:** ^1^Center for Health Economics, Faculty of Humanities and Social Sciences, University of Nottingham, Ningbo, Zhejiang Province, China; ^2^Department of Mathematical Sciences, Faculty of Science and Engineering, University of Nottingham, Ningbo, Zhejiang Province, China; ^3^Yinzhou District Center for Disease Control and Prevention, Ningbo, Zhejiang Province, China; ^4^Ningbo Municipal Center for Disease Control and Prevention, Ningbo, Zhejiang Province, China

**Keywords:** birth weight, overweight, general obesity, central obesity, childhood

## Abstract

**Background:**

Childhood overweight (OW) and obesity (OB) have become a serious public health concern worldwide. The objective of this study is to investigate the association between the levels of birth weight (BW) and OB and central OB in Chinese primary school children.

**Methods:**

A school-based longitudinal study was conducted from 2016 to 2019. Information of children and parents were gained from both children and parents’ questionnaires. Longitudinal anthropometric data were obtained from annual health check. BW (kg) was categorized into 4 groups [<3.0, 3.0–3.4, 3.5–3.9 and ≥ 4.0 (macrosomia)]. Normal weight, OW and OB were defined based on sex-specific and age-specific body mass index (BMI). Central OB was identified using sex-specific waist-to-height ratio (WHtR).

**Results:**

Around 14.5 and 15.6% of 1,204 children had low BW (<3.0 kg) and macrosomia, respectively. The overall rate of OB and central OB at 7–10 years were 10.4 and 28.3%, respectively. Linear-shaped relationships were observed between BW and anthropometric values in both sexes at 7–10 years and 11–13 years. A J-shaped relationship was found between BW and WHtR in boys at 11–13 years. Higher BW status were associated with increased adjusted odds of OB in children (3.5–3.9: OR: 1.5, CI 95%: 1.1–2.0; macrosomia OR: 1.4, CI 95%: 1.0–2.0).

**Conclusion:**

Higher levels of BW (≥ 3.5 kg) were associated with an increased risk of OB in children, but not central OB. The results can support public health specialists for future research and improvement of strategies for childhood obesity prevention.

## Introduction

Childhood overweight (OW) and obesity (OB) is one of the most critical global health concerns in the 21st century (1). Over the past 30 years, OW and OB in children and adolescents worldwide has increased more than 4-fold from 4% in 1975 to 18% in 2016 (2). The short-term effect of childhood OB could be on the child’s physical and psychological comorbidities, and academic attainment (3, 4). The long-term impact of childhood OB is adverse consequences on a higher chance of OB, chronic diseases, premature death and disability in their adulthood (2, 5).

Childhood OW and OB have undergone a rapid growth due to nutrition transition for the past four decades. In China, the prevalence in children and adolescents of OW increased from 1% in 1985 to 13.8% in 2019, and OB increased from 0.1% in 1985 to 9.6% in 2019 (6). In the same period, there were trends where the average birth weight (BW) significantly increased, shifting from low BW (BW < 2.5 kg) to macrosomia (BW ≥ 4.0 kg) (7–9). The incidence of macrosomia in Yantai of Shandong province was reported to be 2.6, 6.9 and 13.2% in the 1970s, 1980s and 1990s, respectively (10). Newborn macrosomia has been documented, gaining more academic attention from clinicians and professional researchers to understand associations with health outcomes in childhood and adulthood (9, 11).

It has been suggested that maternal nutritional status during pregnancy affecting maternal metabolic conditions is playing an important role in determination of fetal nutrition and fetal growth, thus, it is most likely related to BW status, body fat composition of the newborn, and OW and OB in children and adolescents (12). Apart from maternal nutritional and health status, the development of childhood OB can be attributable to complex risk factors including economic boom, urbanization and transitions in dietary patterns and lifestyle, genetics, feeding patterns, parental influence, living environment, technological changes, family socio-economic status and societal concerns (13).

Many epidemiological studies showed that BW was evident to be associated with body mass index (BMI), body composition and the risk of OB in their childhood (14–16) and their adulthood (17) across an individual’s life. Previous cross-sectional and longitudinal studies revealed that high BW was strongly associated with OW and OB (18, 19) and central OB (20, 21) in primary school-children. A cross-sectional study conducted in 31 Chinese provinces suggested that higher levels of BW (≥3.5 kg) were associated with greater risks of developing OB children and adolescents, but lower level of normal BW (2.5–2.9 kg) was associated with a lower risk of OW and OB (22). However, inconsistent results were reported previously (23, 24). One previous report from a Chinese cross-sectional study including 4 municipalities, Hangzhou and Nanjing indicated that J-shaped relationships were observed between BW and BMI z-score, and waist-to-height ratio (WHtR) in childhood (24). Yuan and his colleagues reported that very low BW was associated with central OB in Chinese children and adolescents aged 7–17 years (24).

One recent study conducted in Zhoushan, Zhejiang Province from 2012 to 2021 indicated that infants born with large size at birth for gestational age had a higher risk of OW/OB in early childhood (25). To our best knowledge, the association of BW with childhood OW and OB has not been well studied in Zhejiang Province. As evidence to the existing limited literature, the purpose of the present study is to investigate the association between the levels of BW status and the risk of general OB and central OB in primary school children aged 7–10 years in Ningbo, China. Conducting the present research can utmost provide a comprehensive understanding for prevention of childhood OB in China.

## Methods

### Study design and study population

The Ningbo Youth Risk Behavior Survey (YRBS) was a school-based prospective study conducted in Ningbo, Zhejiang Province, China from October 2016 to October 2019. A multistage, stratified cluster sampling procedure was used to draw the target samples. In total, 22 schools, including 9 primary schools and 13 middle schools, were randomly selected from 10 districts (6 urban, 2 urban–rural junction and 2 rural areas). Invitations were sent to school principals and school management. With permission, grades and classes were randomly selected in each school for this study.

Given Type I error of 0.05 and Type II error of 0.2, the estimated minimum sample size was calculated based on the following formula:


$$n=\left(4\sigma^{2}/{md}^{2}\right)\;\left(1+\left(m–1\right)\;\rho \right)\;{\left(z_{\alpha /2}+z_{\beta }\right)}^{2}$$

Where m = 4 time points, ρ = constant within-subject correlation, σ^2^ = 1 and a difference d = 0.25 at the two-sided 0.05 level. The minimum sample size per group was 770. Considering the follow-up loss rate of 20%, the final estimated sample size was 924.

The selection criteria for children to participate in this study were: (1) children who were born in Ningbo; (2) aged from 6.5 years to 18 years; (3) the children’s signed consent to participate; (4) the written informed consent of a parent or legal guardian for their children’s participation in the study. Children with a disability or an injury, affecting the children’s health examination, were not eligible to participate in the survey. In this present study, children aged 11 years or older were excluded from the baseline study. More details have been described elsewhere (26).

**Figure 1:**
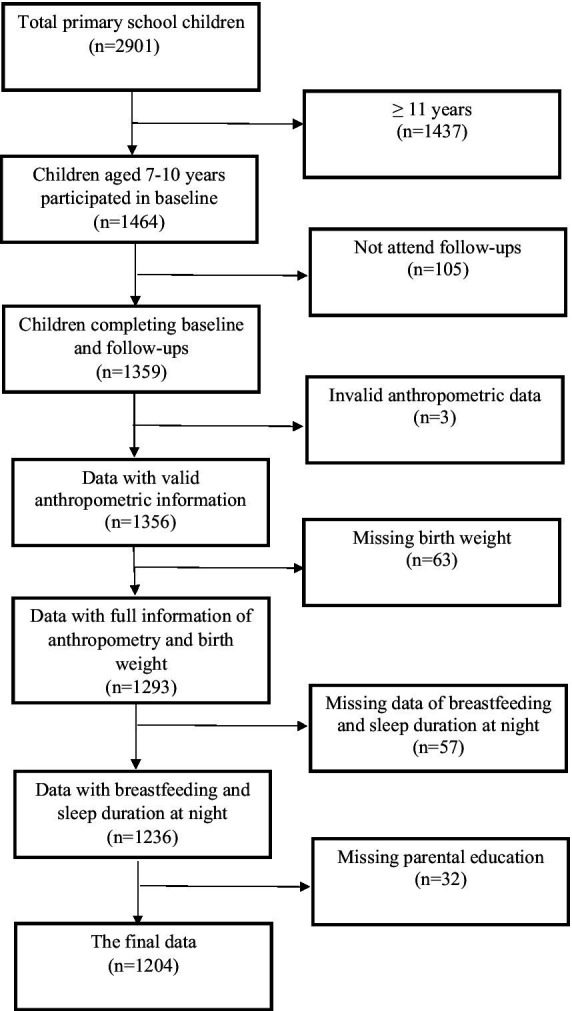
Flowchart of the study population participating in the school-based longitudinal study.

In total, 2,901 children were invited to participate in this study (Figure 1). Due to the age criterion, 1,437 children (49.57%) were excluded. 105 out of 1,464 children did not attend the follow-up surveys. All information was double-checked for quality control during surveys by well-experienced researchers. For missing information, children were asked to re-complete those questions if they were willing and they were able to. After quality control, invalid and missing data were excluded. In the end, 1,204 children were included in the present study.

The present study was conducted according to the guidelines laid down in the Declaration of Helsinki and approved by the ethics committee of the Ningbo Center for Disease Control and Prevention (No. 201703). Written or verbal informed consent was obtained from all schoolchildren and their parent or legal guardian. Verbal consent was witnessed and formally recorded.

### Baseline study

All the eligible children were asked to complete a self-administered standardized questionnaire which was developed based on the YRBS survey in the United States (27). The details of the self-administered standardized questionnaire have been reported previously (26, 28).

All children took part in a health examination in the early morning in the schools. After the health examination, all children filled out the questionnaire within 1 h during their regular class time.

### Follow-up surveys

Follow-up surveys were carried out every year. During the follow-up surveys, children completed the same self-administered standardized questionnaire and had a health examination. In addition to children’s participation, their parent or legal guardian completed a parental questionnaire including parental education, employment status, child’s birth situation and BW. In this present study, we used data and anthropometric variables at 11–13 years in 2019 for comparing with those at 7–10 years in the baseline survey.

### Anthropometric measurements

At the baseline and follow-up surveys, all children were measured in the early morning by well-experienced medical professionals in the schools. All the medical professionals used the same type of medical equipment, which has been described previously (28).

Anthropometric measurements including body weight and height were measured in light clothing and without shoes. Body weight was measured using an electronic scale to the nearest 0.1 kg and height was measured using a free-standing stadiometer to the nearest 0.1 cm (GMCS-I, Xindong Huateng Sports Equipment Co. Ltd., Beijing, China). Waist circumference (WC) was measured at the midpoint between the inferior costal margin and the iliac crest in the midaxillary line.

BMI, identifying general OB henceforth referred to as OB, was calculated following the formula: weight (kg)/height^2^ (m^2^). BMI z-score was calculated to standardize the BMI value across sex and age groups (29). Weight status in Chinese children was classified into normal weight, OW and OB, using sex- and age- specific reference data from National Health and Family Planning Commission of China (30).

Waist-to-height-ratio (WHtR) as a measure of central OB was calculated as WC (cm)/height (cm). Central OB was defined by a sex-specific cut-off value of WHtR according to the definition of metabolic syndrome by Chinese Medical Association (31). WHtR of 0.48 and 0.46 or more defined central OB in boys and girls, respectively.

### Child’s birth weight and breastfeeding duration

Children’s BW (kg) was reported by parents during follow-up surveys. Due to few children with low BW (<2.5 kg), BW was divided into 4 categories: <3.0, 3.0–3.4, 3.5–3.9 and ≥ 4.0 (macrosomia) (24, 32).

Each child’s parent or guardian reported whether the child was breastfed and breastfeeding duration. According to the reports, exclusive breastfeeding duration (months) was classified into 4 groups: no, 1–5, 6 and ≥ 7.

### Socio-economics-status and family structure

Each child’s parents or guardians were asked about their education level during follow-up surveys. The highest degree of maternal and paternal education levels was categorized into three levels: no education or lower secondary education, secondary education and higher education (bachelor or above).

Family structure was gained from children’s reports. Family structure was recorded into three categories: nuclear family, single-parent family and others (e.g., joint family, extended families).

### Statistical analysis

Descriptive analysis was presented as a number and percentage for category variables and mean and standard deviation (SD) for continuous variables based on *p*-value. Statistical differences in mean values and percentages between boys and girls were compared by Student’s t-test and Chi-Square (Χ^2^) test, respectively. Mean anthropometric values across BW categories were tested by ANOVA with Bonferroni correction. As children were randomly selected from different districts and schools, multilevel linear regression was used for multiple-adjusted BMI z-score and multiple-adjusted WHtR across BW categories after adjusting for child’s exclusive breastfeeding status, confounding factors (age and area of residence: urban, urban–rural junction and rural), socio-economics-status (SES; maternal education, paternal education and family structure) and child’s sleep duration at night.

A sensitivity analysis was undertaken for comparison in baseline BMI-z score, WHtR and BW between all children and excluded children aiming to detect whether missing values of excluded children affect the outcomes.

Generalized estimating equations (GEE) with a binary logistic function and an exchangeable correction matrix was used to assess the association between body weight status, and independent variables in the crude model and multivariable model. The GEE model is a method for longitudinal data and allows accounting the correction between OW/OB over time within subjects. Associations were investigated via two models: (1) Model 1: crude model; (2) Model 2: adjusting for child’s breastfeeding status, confounding factors, SES, family structure and sleep duration at night. Interactions were examined between independent variables and confounding factors. Interactions were only retained in Model 2 if they were statistically significant.

Results were considered statistically significant at a two-tailed level of 0.05. Statistical analysis was conducted using the STATA statistical software package version 17 (2021).

## Results

### Study population and characteristics

At baseline, a total of 1,204 children participated in this present study (Figure 1). The overall children’s characteristics and their family at baseline are presented in Table 1. The boys had a significantly higher BW, height and weight than girls. Considering BW status, approximately 14.5% of children had relatively low BW and around 15.6% of children were macrosomia. The results of sensitivity analysis showed no significant difference in children’s baseline BMI-z score, WHtR and BW between all children and excluded children. (BMI-z score: *p* = 0.284; WHtR: *p* = 0.866; BW: *p* = 0.753).

**Table 1:** Characteristics for children at baseline.

	Total (*n* = 1,204)	Boys (*n* = 630)	Girls (*n* = 574)	*p*-value
	n (%)			
Area of residence				0.749
Urban	833 (69.2)	430 (68.3)	403 (70.2)	
Urban–rural junction	265 (22.0)	142 (22.5)	123 (21.4)	
Rural	106 (8.8)	58 (9.2)	48 (8.4)	
Paternal education				0.187
No education or lower secondary education	417 (34.6)	233 (37.0)	184 (32.1)	
Secondary education	330 (27.4)	164 (26.0)	166 (28.9)	
Higher education (bachelor or above)	457 (38.0)	233 (37.0)	224 (39.0)	
Maternal education				0.122
No education or lower secondary education	445 (37.0)	250 (39.7)	195 (34.0)	
Secondary education	313 (26.0)	156 (24.8)	157 (27.4)	
Higher education (bachelor or above)	446 (37.0)	224 (35.6)	222 (38.7)	
Breastfeeding				0.521
No	120 (10.0)	70 (11.1)	50 (8.7)	
1–5 months	295 (24.5)	154 (24.4)	141 (24.6)	
6 months	179 (14.9)	89 (14.1)	90 (15.7)	
≥ 7 months	610 (50.7)	317 (50.3)	293 (51.0)	
Family structure				0.264
Nuclear family	1,088 (90.4)	561 (89.0)	527 (91.8)	
Single-parent family	63 (5.2)	37 (5.9)	26 (4.5)	
Others	53 (4.4)	32 (5.1)	21 (3.7)	
Birth weight status				0.008
<3.0 kg	174 (14.5)	71 (11.3)	103 (17.9)	
3–3.4 kg	475 (39.5)	256 (40.6)	219 (38.2)	
3.5–3.9 kg	367 (30.5)	195 (31.0)	172 (30.0)	
≥4.0 kg	188 (15.6)	108 (17.1)	80 (13.9)	
	Mean (SD)			
Age (years)	8.7 (0.38)	8.7 (0.40)	8.7 (0.36)	0.595
Birthweight (g)	3.5 (0.93)	3.6 (1.0)	3.4 (0.79)	0.006
Weight (kg)	29.6 (8.6)	30.7 (10.4)	28.34 (5.8)	<0.001
Height (cm)	132.8 (6.2)	133.2 (6.1)	132.4 (6.3)	0.040
Duration of sleep at night (hours)	9.6 (0.76)	9.7 (0.80)	9.6 (0.72)	0.309

SD, standard deviation.

The prevalence of OB was significant across body weight status at both baseline (*p* = 0.006) and follow-up (*p* < 0.001; Table 2). The prevalence of OW and OB were 10.1 and 10.4% at baseline, and 12.5 and 6.2% at follow-up, respectively, while the prevalence of central OB at baseline and follow-up were 28.3 and 23.3%, respectively.

**Table 2:** The prevalence of general obesity and central obesity at baseline (7–10 years) and follow-up (11–13 years).

	Total	Boys	Girls	*p*-value
7–10 years				
Body weight status				0.006
Normal	957 (79.5)	479 (76.0)	478 (83.3)	
Overweight	122 (10.1)	72 (11.4)	50 (8.7)	
Obesity	125 (10.4)	79 (12.5)	46 (8.0)	
Central obesity				0.065
No	863 (71.7)	466 (74.0)	397 (69.2)	
Yes	341 (28.3)	164 (26.0)	177 (30.8)	
11–13 years				
Body weight status				<0.001
Normal	978 (81.2)	475 (75.4)	503 (87.6)	
Overweight	151 (12.5)	104 (16.5)	47 (8.2)	
Obesity	75 (6.2)	51 (8.1)	24 (4.2)	
Central obesity				0.077
No	923 (76.7)	470 (74.6)	453 (78.9)	
Yes	281 (23.3)	160 (25.4)	121 (21.1)	

### Relationships between birth weight and anthropometric data

**Figure 2:**
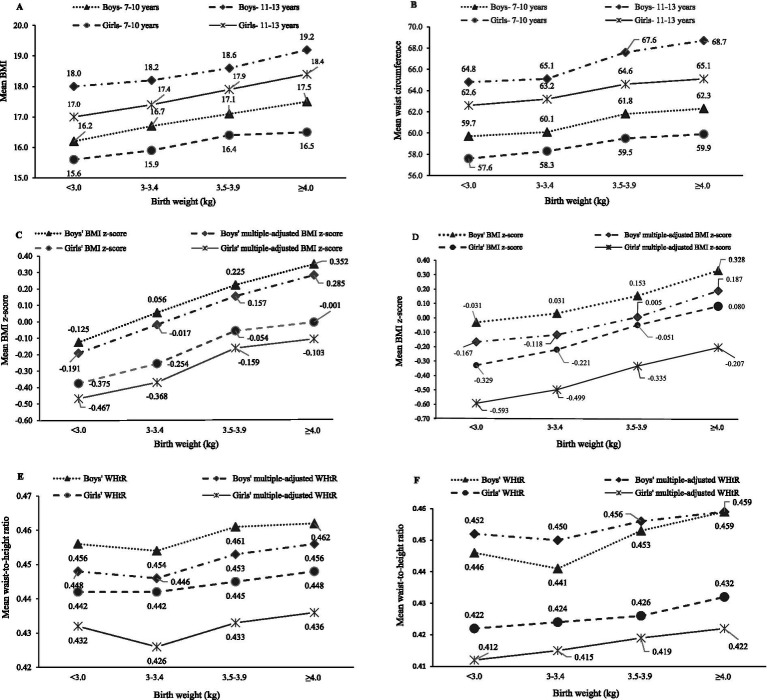
Sex difference in mean anthropometric values across birth weight status (kg) at baseline (7–10 years) and follow-up (11–13 years). Multiple-adjusted BMI and multiple-adjusted WHtR were adjusted for child’s breastfeeding status, age, area of residence, maternal education, paternal education, family structure and sleep duration at night. **(A)** 7-10 years and 11-13 years; **(B)** 7-10 years and 11-13 years; **(C)** 7-10 years; **(D)** 11-13 years; **(E)** 7-10 years; **(F)** 11-13 years.

Mean anthropometric data was compared in children at 7–10 and 11–13 years across BW status (Table 3; Figure 2). Mean BMI, BMI z-score, WC and WHtR of the total children significantly increased across BW status at both baseline and follow-up, with the exception of multiple-adjusted WHtR. Based on descriptive analysis, linear-shaped relationships were found between BW and BMI, BMI z-score, multiple-adjusted BMI z-score and WC in boys and girls at 7–10 and 11–13 years (*p* < 0.05 for all the sex and age groups). There was a J-shaped relationship of BW with WHtR in boys at 11–13 years (*p* = 0.026).

**Table 3:** Mean anthropometric values with standard deviation in children at baseline (7–10 years) and follow-up (11–13 years) based on birth weight status.

	Birth weight (kg)				*p*-value for trend
	<3.0 (*n* = 174)	3–3.4 (*n* = 475)	3.5–3.9 (*n* = 367)	≥4.0 (*n* = 188)	
7–10 years					
BMI	15.8 (2.2)	16.3 (2.5)	16.8 (2.7)^a^^,^^d^	17.1 (2.7)^a^^,^^d^	<0.001
BMI z-score	−0.273 (0 0.87)	−0.087 (0.95)	0.094 (1.0)^a^^,^^d^	0.201 (1.0)^a^^,^^d^	<0.001
Multiple-adjusted BMI z-score*	−0.324 (1.3)	−0.189 (1.7)	−0.000 (1.6)	0.091 (1.3)	<0.001
WC	58.5 (6.4)	59.3 (6.8)	60.7 (7.3)^b^^,^^d^	61.3 (7.4)^a^^,^^d^	<0.001
WHtR	0.448 (0.04)	0.448 (0.05)	0.453 (0.05)	0.456 (0.05)	0.126
Multiple-adjusted WHtR*	0.440 (0.06)	0.438 (0.08)	0.443 (0.07)	0.446 (0.06)	0.155
11–13 years					
BMI	17.4 (3.4)	17.8 (3.1)	18.3 (3.3)^b^	18.9 (3.6)^a^^,^^d^	<0.001
BMI z-score	−0.207 (1.0)	−0.086 (0.93)	0.058 (0.97)^b^	0.223 (1.1)^a^^,^^d^	<0.001
Multiple-adjusted BMI z-score*	−0.387 (2.0)	−0.301 (3.0)	−0.164 (2.7)	−0.004 (2.0)^a^	<0.001
WC	63.5 (8.4)	64.2 (8.4)	66.2 (8.7)^b^^,^^d^	67.2 (8.9)^a^^,^^c^	<0.001
WHtR	0.432 (0.05)	0.433 (0.05)	0.440 (0.05)	0.447 (0.06)^b^^,^^d^	0.007
Multiple-adjusted WHtR*	0.4396 (0.09)	0.4374 (0.14)	0.442 (0.13)	0.445 (0.09)	0.199

BMI, body mass index; WC, waist circumference; WHtR, waist-to-height ratio. *Adjusted for child’s breastfeeding status, sex, area of residence, maternal education, paternal education, family structure and duration of sleep at night.

a Mean value was significantly different from birth weight < 3.0 kg, *p* ≤ 0.001.

b Mean value was significantly different from birth weight < 3.0 kg, *p* < 0.05.

c Mean value was significantly different from birth weight 3–3.4 kg, *p* ≤ 0.001.

d Mean value was significantly different from birth weight 3–3.4 kg, *p* < 0.05.

### Associations between birth weight and the risk of obesity and central obesity

Odds ratio (OR) derived from multivariable GEE analysis showed that children’s BW with 3.5–3.9 kg and ≥ 4.0 kg was only significantly associated with higher odds of children’s OB in Model 1 (3.5–3.9: OR = 1.4, 95% CI: 1.1–1.9; ≥4.0: OR = 1.5, 95% CI: 1.1–2.1; Table 4). After adjusting for child’s breastfeeding status, confounding factors, SES and sleep duration at night, higher children’s BW remained to be associated with increased odds of developing children’s OB (3.5–3.9: OR = 1.5, 95% CI: 1.1–2.0; ≥4.0: OR = 1.4, 95% CI: 1.0–2.0). In the adjusted analysis, girls were associated with lower odds of OB compared to boys (OR = 0.534, 95% CI: 0.414–0.687). Regarding central OB in children, central OB was insignificantly associated with children’s BW status in both sexes.

**Table 4:** Odds ratios for overweight, general obesity and central obesity in primary school children based on birth weight status.

	Birth weight (kg)								*p*-value for trend
	<3.0 (*n* = 174)		3–3.4 (*n* = 475)		3.5–3.9 (*n* = 367)		≥4.0 (*n* = 188)		
	OR	95% CI	OR	95% CI	OR	95% CI	OR	95% CI	
Overweight/obesity									
Crude model	0.716	0.473–1.1	1		1.4	1.1–1.9	1.5	1.1–2.1	<0.001
Adjusted model*	0.753	0.495–1.1	1		1.5	1.1–2.0	1.4	1.02–2.0	<0.001
Central obesity			1						
Crude model	0.753	0.495–1.1	1		1.5	1.1–2.0	1.4	1.01–2.0	0.150
Adjusted model*	0.966	0.712–1.3	1		1.2	0.925–1.5	1.2	0.913–1.7	0.392

CI, confidence interval; OR, odds ratio. *Adjusted for child’s breastfeeding status, sex, age, area of residence, maternal education, paternal education, family structure and duration of sleep at night.

## Discussion

Our results showed positive linear-shaped relationships between BW and anthropometric values in both boys and girls at both 7–10 and 11–13 years, and a J-shaped relationship between BW and WHtR in boys at 11–13 years. The results derived from GEE analysis indicated that higher levels of children’s BW (≥3.5 kg) were associated with increased odds of OW and OB in children, but not central OB.

With economic growth, the changes in feeding culture and dietary patterns during pregnancy has a critical effect on BW status (12). Mean BW (3.5 kg) and the prevalence of macrosomia (15.6%) in our study were relatively higher than those of Chinese children derived from previous cross-sectional studies (24, 32, 33). However, the rates of low BW (<3 kg) was lower than previous reports (24, 32). The possible reason may be attributable to the study year and study areas. The prevalence of OW/OB at both 7–10 years (20.5%) in our study were higher than those children at 7–10 years living in eastern China (around 15–18%) from 2017 to 2019, but the prevalence at 11–13 years (18.7%) in our study were close to that of children at 11–13 years living in eastern China (ranging from 16 to 18%) (34). Consistent with our findings, the prevalence of OB was higher in boys (7–10 years: 10–12%; 11–13 years: 8–11%) than girls (7–10 years: 7–8%; 11–13 years: 6–8%) (34). In addition, the prevalence of OW/OB in our study was similar to the reports of children and adolescents from a previous Chinese cross-sectional study including 4 municipalities and 2 provincial capitals (20.3%), but lower than the national level of OW/OB (23.4%) in Chinese adolescents aged 7–18 years in 2019 (6, 24). We noticed that the prevalence of central OB in our study at both 7–10 years (28.3%) and 11–13 years (23.3%) were much higher than that of Chinese children (21, 24). Comparing to childhood OB in western countries, our results were extremely lower than children in the United States in 2020 (6–11 year: 20.7%, 12–19 years: 22.2%) (35) and European children aged 7–9 years in 2017 (36).

BW is known as a key factor of OB during childhood (37). The relationship between BW and childhood OB has been investigated in many epidemiological studies. Most studies have found positive association between BW and the risk of OB, and central OB in school-age children (15, 21, 38, 39). In our study, a J-shaped relationship was found between BW and WHtR in boys at 11–13 years and a linear-shaped relationship was found between BW and BMI z-score in both boys and girls at 7–10 years and 11–13 years, which is consistent with some reports from large population-based studies (21, 24). However, a U-shaped association between BW and the risk of OB was found in some previous studies (23, 24). Due to few cases of low BW (<2.5 kg) in our study, the classification of BW might result in a different shaped relationship with BMI z-score.

BMI is a measure for indicating nutritional status, which cannot determine distribution of body adiposity. However, WHtR can reflect central adiposity which is suggested to be associated with cardio-metabolic diseases (40). In our study, BW was a strong risk factor to develop childhood OB, but not central OB. A meta-analysis of case–control studies indicated that high BW (≥ 4 kg) was associated with increased risk of OB in Chinese children (41). Few studies have investigated both OB and central OB in primary school-age children. A cross-sectional study elaborated that macrosomia was positively associated with OB (≥4.5 kg) and central OB (4.0–4.4 kg) in Chinese children and adolescents, and low BW (<1.5 kg) was only associated with the risk for central OB (24). Inconsistent findings derived from a school-based epidemiology survey showed higher BW (≥3.5 kg) were associated with increased risk of central OB in Chinese children and adolescents (7–17 years), but not low BW (21). In agreement with our findings, a case–control study conducted on Cuban children aged 7–11 years revealed that BW is not a risk factor for central OB in children aged 7–11 years (38). Age is an important factor for childhood central OB, which has been proven by the reports derived from a large-population based cross-sectional survey (21). Yang et al. found that the risk of central OB was only observed in BW (< 4 kg) in those of younger ages (6–9 years), not adolescents (21). The possible reason for this difference might be relatively small sample size of our study, although the statistical power has reached 80%. It is well known that the changes in body fat distribution and regional fat mass start during puberty because of hormones including cortisol, growth hormone and sex steroids (42, 43). The difference in body composition and body fat distribution between children and adolescents could explain the discrepancy of risks between OB and central OB in our study.

Many epidemiological studies have confirmed that higher BW categories are most likely to develop OB (21, 23, 24, 32) and central OB (21, 24) later in childhood. Our findings showed higher BW status (≥3.5 kg) was significantly associated with higher odds of OB, in particular for boys. Six out of seven studies included in a systematic review and meta-analysis showed strongly positive associations between high BW and childhood OB (37). In agreement with our findings, children with higher BW (≥3.5 kg) had tended to be OB, compared to normal BW (23, 24), in particular for those that had macrosomia. A meta-analysis including 66 studies from 26 countries showed that macrosomia was positively associated with increased long-term risk of childhood OB compared with normal BW (2.5–4.0 kg) (44). The risk of childhood OB for macrosomic infants under 3 years was 3.74 and 1.64 times, based on weight-for-age and BMI-for-age, respectively higher than children with normal BW (45). The mechanism of the relationship between higher BW/macrosomia and childhood OB can be that a large fetus is affected by higher maternal lipogenesis and increased fatty acid and glucose transporters to fetal adipocytes due to overfeeding during pregnancy (46). This can be associated with higher BW and body composition, which may determine enlargement of adipose cell size and have a great number of adipose cells in early life, and have long-term effects during childhood (46, 47).

Interestingly, we noticed that low BW (< 3 kg) was relatively associated with higher WHtR in both boys at both age groups and girls at 7–10 years compared to normal BW (3–3.4 kg). A recent systematic review and meta-analysis showed the association of low BW with insulin sensitivity in childhood and adolescence (48). In line with our findings, many studies indicated that the existence of low BW (<2.5 kg) increased the risk of childhood OB (21, 24, 49). However, some studies showed the inconsistent results that low BW was not a risk factor for OB (32, 44). Yuan et al. indicated that children with only very low BW (< 1.5 kg) had the highest risk of central OB (24). The mechanism is unclear. The possible reason might be that childhood OB is attributed to long-term adverse effects because of catch-up growth.

The association between BW and childhood OB is influenced by many factors including environment, genetics and lifestyle. In this study, we further analyzed sex showing boys had higher risk of OB across BW categories, including low BW and high BW, compared to girls. The higher risk of OB in boys than girls is attributable to the biological differences in body composition during the fetal and infant growth (50). In addition, puberty at early adolescence may have a greater impact on increased fat content in the central region in boys compared to girls, which could cause android shape in boys (42). This may account for why only a J-shaped relationship was found in boys at 11–13 years in our study.

This school-based longitudinal study was the first study to investigate BW and OB and central OB in children aged 7–10 years with a 3-year follow-up in Zhejiang Province with standardized children’s and parental questionnaires along with annual health examinations by well-experienced nurses and medical doctors. Although the significant findings can provide evidence of associations between BW and childhood OW/OB to fill the gap of the currently limited literature in Zhejiang Province, this study still has several limitations. Firstly, children’s BW was obtained from a parental questionnaire instead of hospitals, which relied on parental memory. However, these parental-reported BW was almost consistent with their children’s report. Secondly, children’s birth length and growth pattern when under 5 years old were not collected. These factors were not fully controlled for the association of BW with the risk of OB and central OB, which might affect our final outcomes. In addition, parental occupation and household income were not collected in this present study, which might influence the accuracy of the association between BW and childhood OW/OB and central OB. Last but not least is that parental weight status and health status (e.g., cardio-metabolic diseases) during pregnancy were not collected. It is known that parental health status, especially for the mother, could lead to higher BW/macrosomia. More well-designed longitudinal studies with precise physical examination, complete information at birth and parental nutrition status during pregnancy are needed to explore how high/low BW is associated with OB and central OB in Chinese children and adolescents, and its mechanism.

## Conclusion

Linear-shaped relationships were found between BW and anthropometric values in both sexes 7–10 and 11–13 years, and a J-shaped relationship was observed between BW and WHtR in boys at 11–13 years. Our results indicated that BW was a critical predictor of OB in Chinese school-age children, but not central OB. Higher BW (≥ 3.5 kg) was associated with increased odds of OW and OB in children. Our findings can contribute to the better understanding of the role of BW associated with childhood OB and assist public health specialists and clinicians for improvement of strategies for early intervention against childhood OB.

## Data Availability

The data is not publicly available due to privacy or ethical restrictions. If there is a reasonable request, it can be obtained from the corresponding authors.
